# Hungarian Structural Focus: Accessibility to Focused Elements and Their Alternatives in Working Memory and Delayed Recognition Memory

**DOI:** 10.3389/fpsyg.2021.514886

**Published:** 2021-04-27

**Authors:** Tamás Káldi, Ágnes Szöllösi, Anna Babarczy

**Affiliations:** ^1^Institute for General and Hungarian Linguistics, Hungarian Research Centre for Linguistics, Budapest, Hungary; ^2^Institute for the Methodology of Special Needs Education and Rehabilitation, Bárczi Gusztáv Faculty of Special Needs Education, Eötvös Loránd University, Budapest, Hungary; ^3^Department of Cognitive Science, Faculty of Natural Sciences, Budapest University of Technology and Economics, Budapest, Hungary; ^4^Institute of Cognitive Neuroscience and Psychology, Research Centre for Natural Sciences, Budapest, Hungary

**Keywords:** linguistic focus, memory accessibility, representation, probe recognition, working memory, delayed recognition

## Abstract

The present work investigates the memory accessibility of linguistically focused elements and the representation of the alternatives for these elements (i.e., their possible replacements) in Working Memory (WM) and in delayed recognition memory in the case of the Hungarian pre-verbal focus construction (preVf). In two probe recognition experiments we presented preVf and corresponding focusless neutral sentences embedded in five-sentence stories. Stories were followed by the presentation of sentence probes in one of three conditions: (i) the probe was identical to the original sentence in the story, (ii) the focused word (i.e., target) was replaced by a semantically related word and (iii) the target word was replaced by a semantically unrelated but contextually suitable word. In Experiment 1, probes were presented immediately after the stories measuring WM performance, while in Experiment 2, blocks of six stories were presented and sentences were probed with a 2-minute delay measuring delayed recognition memory performance. Results revealed an advantage of the focused element in immediate but not in delayed retrieval. We found no effect of sentence type on the recognition of the two different probe types in WM performance. However, results pertaining to the memory accessibility of focus alternatives in delayed retrieval showed an interference effect resulting in a lower memory performance. We conclude that this effect is indirect evidence for the enhanced activation of focus alternatives. The present work is novel in two respects. First, no study has been conducted on the memory representation of focused elements and their alternatives in the case of the structurally marked Hungarian pre-verbal focus construction. Second, to our knowledge, this is the first study that investigates the focus representation accounts for WM and delayed recognition memory using the same stimuli and same measured variables. Since both experiments used exactly the same stimulus set, and they only differed in terms of the timing of recognition probes, the principle of ceteris paribus fully applied with respect to how we addressed our research question regarding the two different memory systems.

## Introduction

The present work investigates the memory accessibility of linguistically focused elements in Hungarian and their representation in Working Memory (WM) and with a delay before retrieval. There are a multitude of theories regarding how focused elements and their alternatives are represented in memory predicting contradictory outcomes. Therefore, the primary aim of the present work is to further investigate the issue at hand, and to offer an explanation for findings based on general psychological principles pertaining to human memory. Since currently there is no data regarding the memory representation of the focused element and its alternatives for Hungarian focus, a secondary aim is to fill this gap by investigating how this particular focus construction affects WM performance, and memory performance when one is not able to rely exclusively on processes for maintaining information in WM (i.e., in a delayed recognition memory task). A tertiary aim is to investigate what can potentially belong to the set of alternatives evoked by focus.

Regarding these issues, we formulated and tested the following predictions. We predicted that focused elements are more readily accessible in WM than corresponding non-focused elements. Since results on delayed retrieval are scarce in the literature, we made no predictions regarding the accessibility to focused elements when there is a delay before retrieval, and hence one can not rely on WM processes. However, we made the tentative suggestion that the facilitatory effect observed in WM disappears. As far as focus alternatives are concerned, earlier results are contradictory, therefore, we aimed to adjudicate between the two conflicting predictions that focus enhances the representation of focus alternatives or it does not. Regarding the question of what constitutes the set of focus alternatives, we tested the prediction that—if alternatives are generated at all, not only semantically but contextually related alternatives are also activated.

### Functional Characterizations of Linguistic Focus

Linguistic focus is an information packaging device (Chafe, 1976; Krifka, 2008) which pertains to “the information in the sentence that is assumed by the speaker not to be shared by him and the hearer” (Jackendoff, 1972, p. 16), hence focus expresses new, non-presupposed information (see also Kiss, 1998). In generative linguistic frameworks, focusation is often analyzed as movement to a functional projection. An interesting work from this domain suggests that certain movements or extraction phenomena (like movements from islands) may be related to a principle called semantic dominance (Erteschik-Shir, 1973) or dominance (Erteschik-Shir and Lappin, 1979). According to the principle of dominance, “a constituent c of a sentence S is dominant in S if the speaker intends to direct the attention of his hearers to the intension of c, by uttering S” (Erteschik-Shir and Lappin, 1979, p. 43). This principle clearly predicts that a to be focused element should appear in a designated syntactic focus position if the adopted theoretical linguistic framework assumes that sentences are derived and that derivations involve movements. Positing movements, together with the corollary that traces are left behind, gives rise to a number of psycholinguistic questions regarding how these structures are processed and represented. Since these questions are beyond the scope of the present work, we will confine our investigation to the memory representation of focused elements and their alternatives without committing ourselves to any formal theory of syntax on focus. There are two central functional characterizations of focus in the literature.

First, it is claimed that the function of focus is to partition the sentence into two parts: the foreground and the background (see e.g., von Stechow, 1991; Krifka, 1992). Focus is claimed to mark the foreground, highlighting important, emphatic, interesting, contrasted or new information against the background, which is often but not necessarily taken to be part of the common ground of the interlocutors.

Second, based on Rooth's alternative semantics approach (Rooth, 1985, 1992), it is commonly held that the function of focus is to evoke a set of alternatives: it expresses that there is a set of entities whose subset is selected by focus. Both functional definitions, namely the function of highlighting against a background and the function of signaling the presence of alternatives have been taken up by psycholinguistic enquiry and shown to have psychological reality.

### Psycholinguistic Results on the Functional Aspects of Focus

The highlighting function of focus has been related to attention in the psychology of language processing: a wide array of studies have shown that the psychological function of focus is to guide the attention of the listener to the focused element. For example, in a seminal paper Bredart and Modolo (1988) used it-cleft constructions, a type of syntactic focus, to investigate whether the so-called Moses illusion is modulated by focus. The authors presented anomalous cleft sentences, such as *It was Moses who took two animals of each kind on the Ark*. The sentence is anomalous, since according to the Biblical story, it was Noah who brought two animals of each kind onto the ark before the flood. Participants were instructed to carry out a sentence verification task. The variable of interest was how frequently participants spotted the anomaly as a function of whether the incongruous item (i.e., *Moses* in the above example) was focused or unfocused. The results indicated a higher detection rate in the focused condition lending support to the idea that focus indeed guides attention to the focused element.

Relying on the findings of Bredart and Modolo (1988) and Sturt et al. (2004) investigated how the level of detail with which a word is represented in the mind is modulated by focus. The authors hypothesized that since focus directs attention to the focused element, this element is subject to in-depth processing, and consequently its representation is more fine-grained than those of unfocused elements. Sturt et al. (2004) tested this hypothesis using a change detection paradigm in which participants read short texts containing a cleft sentence in which the target word was either focused or not. Critical probes were the same texts containing one change: the target word was either replaced by a semantically related word, or a semantically unrelated word. The results revealed that the detection rates were equally high irrespective of focus in the unrelated condition. However, in the semantically related condition, focus made a difference: while changes were significantly less likely to be detected when the critical noun was not focused, detection rates remained high in the related condition when it was in focus. Based on these results, the authors concluded that focus indeed directs attention and thus can modulate the specificity or granularity of the meaning representation of linguistic expressions (on granularity see Hobbs, 1990). This account of focus representation was named the granularity account by Sanford et al. (2006) and has been tested by a number of subsequent studies with confirmatory results (see e.g., Sanford et al., 2006, 2009; Ward and Sturt, 2007).

Another approach to the highlighting function of focus is the identification account formulated by Almor and Eimas (2008). This account proposes that the primary function of focus is to facilitate the identification of the focused element in order to enhance the efficiency of the discourse integration of linguistic elements. To test this hypothesis, Almor and Eimas (2008) investigated how syntactic focus (i.e., the cleft construction) modulated the accessibility of linguistic elements immediately after the focus-containing sentence has been processed in a lexical decision paradigm using reaction time (RT) as a dependent variable. The results showed that participants responded faster when the antecedent of the subject was focused compared to when it was not, lending support to the hypothesis that focused elements are more accessible in online processing. Almor and Eimas (2008) also investigated the long-term accessibility of the focused elements using a questionnaire in which questions elicited the delayed recall of critical focused words. In the recall task, the authors found an adverse effect for focus: if the critical word was marked for focus earlier during the experiment, its recall rate was lower compared to when it was unfocused.

In sum, the results of experimental work on the highlighting or attention capturing function of focus inspired the formulation of two mutually non-exclusive accounts of focus representation: the identification account and the granularity account. Note, that the granularity account is a stronger one: it includes the predictions of the identification account, since it claims that focus has an attention capturing property: if a linguistic element captures the attention of the addressee, its identification will also be fostered. Furthermore, the granularity account claims that focus leads to an in-depth processing of the focused element resulting in a more fine grained representation. For these reasons, we abandon the identification account, and test the predictions of the granularity account in the present work.

The strand of research inspired by the alternative semantics approach to focus (Rooth, 1985, 1992; Krifka, 1992, 2008) concentrates on the activation of alternatives generated by a focus containing expression. An account formulated in this vein is the so-called contrast account (see e.g., Braun and Tagliapietra, 2010; Fraundorf et al., 2010), which proposes that in the case of contrastive focus, the contrast set of the focused elements receives a higher activation with respect to semantically related, but not necessarily contrasted elements, or to unrelated elements.

For example, testing the contrast account, Fraundorf et al. (2010) investigated the accessibility of the contrast set generated by test sentences with a contrastive accent (L + H^*^ accent) as opposed to sentences with a non-contrastive accent (H^*^ accent associated with new, non-contrasted information) using a delayed forced choice recognition task on the target words. The results showed improved performance on the contrastively accented words relative to the words with non-contrastive accent. Fraundorf et al. (2010) concluded that the observed long-term effect rules out the identification account, but it is compatible with both the granularity account and the contrast account. To tease apart these accounts, the authors carried out a sentence verification experiment using the same materials. In this experiment participants were presented statements in three conditions and had to verify their truth with respect to the sentences heard earlier. Statements belonging to the three conditions (i) contained the same target item as the test sentence, (ii) contained a mentioned contrast item, or (iii) contained an unmentioned but within-category item. The granularity account predicts an enhanced representation primarily for the focused item, whereas the contrast account predicts that the representation of both the focused item and the members of its contrast set should be enhanced. Therefore, Fraundorf et al. (2010) argue that if the former account is tenable, no advantage should be observed for either the mentioned contrast item or the unmentioned alternative. According to the contrast account, however, the sentence containing the mentioned contrast item should be enhanced while the advantage should not extend to the sentence containing the unmentioned alternative, since the unmentioned item was not a member of the original contrast set. The results were found to support the contrast account.

Another account using Rooth's (1985) alternative semantics as a point of departure is the focus association account, which proposes that alternatives for focus are enhanced whether they are in the contrast set or not. In one experiment by Gotzner et al. (2013) the authors compared the accessibility of prosodically focused elements using the contrastive L+H^*^ accent used also by Fraundorf et al. (2010) and elements marked for focus by the particles *only* (*nur*) or *also* (*auch*) together with the L+H^*^ contrastive accent. Participants performed a probe recognition task after hearing stories as in (1) (contrastive accent marked by capital letters).

(1) Context sentence: The judge and the witness followed the argument.
Critical sentence: (Only/also) the [JUDGE]_Focus_/the [judge]_Focus_ believed the defendant.
Extra filler sentence: He announced the verdict.

After the story, a probe word was presented which was the mentioned alternative in the context sentence of the critical conditions (*witness*). The task of the participants was to decide if the word had appeared in the story or not. The results revealed that RTs were fastest in the contrastive accent condition indicating that the accessibility of alternatives was enhanced by contrastive focusing. However, inclusion of the focus particles resulted in longer RTs, which, as the author argues, is the consequence of interference: if focus is explicitly used to mark the presence of alternatives (as in the case of *only* and *also*), the focus alternatives become more activated. According to the authors, this higher activation of the set of alternatives in turn led to a greater level of competition during the probe recognition task manifest in an interference effect, i.e., in longer RTs when focus particles were used together with contrastive accent.

In another experiment, Spalek et al. (2014) presented stories containing sentences marked for focus by particles (*nur* ~ *only* and *sogar* ~ *even*) in blocks of ten, and investigated the memory accessibility of alternatives using a delayed recall task. The results revealed that while there was no facilitative effect of focus particles on the recall of the focused elements themselves, the presence of a particle significantly increased the recognition rate of the focus alternatives.

The rather selective summary of experimental studies above reveals that there is considerable diversity in the methods of inquiry, the investigated focus types (within and across languages), the findings, and also in the theories of focus representation. Note that findings pertaining to the memory representation of focused elements and alternatives come both from tests given immediately after the presentation of the critical sentence (e.g., Bredart and Modolo, 1988; Sturt et al., 2004; Ward and Sturt, 2007; Almor and Eimas, 2008; Gotzner and Spalek, 2016), and tests given after a delay of a few seconds (e.g., Almor and Eimas, 2008; Spalek et al., 2014). Authors in the field argue that these results reflect the interaction of focusing with two separate memory systems: Working Memory (WM) and Long-Term Memory (LTM). WM is the cognitive system responsible for storing, processing and manipulating information needed for a given cognitive task for a limited period of time (Baddeley, 2003, 2009; Unsworth and Engle, 2007; Cowan, 2008), while LTM is responsible for storing information over long periods of time (Cowan, 2008; Baddeley, 2009).

Apart from issues related to the representation and accessibility of focus alternatives, the question of what constitutes this set has also been taken up by psycholinguistic research.

As fleshed out by Gotzner (2017), there is a permissive and a restrictive view. The permissive view, based on Rooth (1992), claims that it is the context that serves to designate the alternative set, therefore, alternatives are selected based on pragmatic principles. On the other hand, the restrictive view claims that only those elements can constitute such a set that are semantically contrasted (Wagner, 2006). Consider example (2) (adopted from Wagner, 2006).

(2) a. He produces high-end convertibles. What did he bring as a present to the wedding?
b. He brought a [cheap]_Focus_ convertible.
c. ^*^ He brought a [blue]_Focus_ convertible.

According to the restrictive view, since color has no relation to quality or cheapness, being blue (2c) cannot constitute an alternative to being high-end, as being cheap can (2b). In order to test the predictions of the permissive and restrictive views, Gotzner (2017) re-analyzed data from a lexical decision experiment (Gotzner et al., 2016) by categorizing the stimuli into two groups. In one group (replacement group) the unmentioned probe could be a potential replacement of the focused element in the test sentence (test sentence: *He only bought jackets*, related probe: *trousers*, unrelated but possible probe: *lychees*). The other group contained trials in which the unmentioned probe could not be a possible replacement of the focused element (test sentence: *He only caught flies*, unrelated and impossible probe: *sofas*). Including the factor of Replacement in the analysis revealed that responses for semantically unrelated but possible replacements and unmentioned but semantically related items were equally fast leading to the conclusion that unrelated items can be a part of the set of alternatives if they are possible replacements.

In a subsequent study Jördens et al. (2020) investigated the activation of contextually suitable but taxonomically different alternatives in a cross-modal priming paradigm experiment with probe recognition. Participants were presented with sentences (e.g., *The farmer brought straw into the barn*) in which either the element to be probed (i.e., prime word, e.g., *straw*) was marked for focus by accent or another element (e.g., *farmer*). Two types of probes were presented: one type was either a contextually related and potential focus alternative to the prime word (e.g., *cow* when *straw* is focused) or it was related to the sentence, but not a potential alternative (e.g., *cow* when *farmer* was in focus). The other probe type was both semantically unrelated and contextually inappropriate (e.g., *elevators* with respect to the example sentence above). The experimental task was to indicate whether the probe had appeared in the sentence. RT data revealed that participants were fastest responding to unrelated probes, most probably due to their marked deviation from the prime words. More interestingly, RTs measured for potential alternatives were faster than for inappropriate alternatives indicating a higher activation level for the former probe type. Thus, Jördens et al. (2020) concluded that the set of alternatives that focus generates is contextually determined. The findings of Gotzner (2017) and Jördens et al. (2020) support the permissive view of the generation of focus alternatives.

Hypotheses derived from the accounts mentioned so far can be summed up as follows. Regarding the short-term effects of focus on the representation of the focused element, all accounts make the same claim: the representation of the focused element is enhanced. Hypotheses regarding the activation of focus alternatives do not entirely diverge either. The granularity account is not explicit regarding this question, however, it makes it possible to derive a hypothesis about alternatives: since the focused element has a finer grained representation (Fraundorf et al., 2010), we can expect that semantically related alternatives may be rejected more readily. Note, however, that this is expected as a consequence of the high activation and detailed representation of the representation associated with the focused element itself. In contrast, the focus association account makes the explicit hypothesis that the representation of focus alternatives is enhanced. Furthermore, the permissive view on focus alternatives suggests similar activation levels for semantically and contextually related alternatives, while the restrictive view claims that facilitation should only be observed in the case of semantically related alternatives.

As mentioned earlier, few studies have investigated the memory accessibility of the focused element and its alternatives when there is a delay before retrieval (when the task cannot be completed by involving only WM processes). With respect to such so-called long-term effects, theories on the representation of both the focused element and its alternatives diverge. Regarding the focused element, the granularity and contrast accounts hypothesize that its representation is enhanced, while the identification and focus association accounts do not make any specific claim on this matter. Note that when testing the identification account, Almor and Eimas (2008) found an adverse effect which was explained in terms of the repeated name penalty. The repeated name penalty is an adverse effect on processing that occurs when a referring NP is repeated in consecutive sentences. It does not occur when an anaphor is used in the second sentence. Since in our experiment there were no repeated names, this explanation is irrelevant here. Note also that although the focus association account does not make any specific claim about the representation of the focused element, testing this account, Spalek et al. (2014) found no facilitation in the case of German focus particles *nur* (*only*) and *sogar* (*even*).

Regarding focus alternatives, the identification account along with the granularity account does not make a claim regarding the activation level of these items. On the other hand, the contrast and focus association accounts claim that there is an enhancement in the representation of alternatives.

### The Hungarian Pre-verbal Focus

In Hungarian, focus is simultaneously marked syntactically and prosodically. As exemplified in (3a), the focused element is situated pre-verbally while if present, the verbal modifier (VM) occupies a post-verbal position. Also, the focused element carries a so-called eradicating stress, i.e., it bears the most prominent sentential stress deleting all consecutive stresses within the following sentential domain (Kornai and Kálmán, 1988). Since the pre-verbal position exemplified in (3a) is strictly associated with focus, we will refer to this sentence type as pre-verbal focus (preVf). In focusless, neutral sentences such as (3b), however, it is the VM that occupies the pre-verbal position, while the element corresponding to focus in (3a) sits in a neutral post-verbal position.

(3) a. Miki [egy'tányért]_Focus_ rakott be a szekrénybe.
  Mike [a plate]_Focus_ put into_VM_ the cupboard-in
  Miki put a plate in the cupboard.
b. Mike be-rakott egy tányért a szekrénybe.
  Miki into_VM_-put a plate the cupboard-in
  Mike put a plate in the cupboard.

The function of identification and highlighting, as well as the function of evoking alternatives have also been discussed with respect to preVf in the theoretical literature.

Concerning the foregrounding or highlighting function of focus, Brassai made influential observations already in the middle of the nineteenth century. The author divided the sentence into two parts and claimed that the elements in the part that we today identify as focus “practically lay a basis for the meaning of the sentence in the listener's mind, i.e., they are calling attention, and pointing forward, connecting the mental activity of the listener with that of the speaker” (1860, p. 341; translation by Kiss, 2008, p. 55). This psychological and functional definition is especially appealing, since it is exactly in line with the literature on the attention capturing properties of focus.

Regarding the function of evoking an alternative set, Kenesei (2006), in the vein of alternative semantics of Rooth (1985) and Roberts (1998), proposes that preVf selects a proper subset of a contextually available set, therefore inevitably creating a complementary set containing focus alternatives. The author adds that as a consequence of this property, preVf is necessarily contrastive. Other authors take a more permissive approach regarding the contrastive nature of preVf, and claim that it is only contrastive if it operates on a closed set of (contextually defined) elements (Kiss, 1998). This stance is compatible with the more general formulation of the contrastive function of focus by Krifka (2008), who claims that contrast is only present if the alternatives are directly mentioned and contrasted in a corrective or additive way.

One empirical work studied the relation of preVf and its function of evoking sets. Káldi et al. (2020) examined the contextual effects that trigger the use of preVf in a semi-guided production study. Results revealed that preVf is produced reliably in contexts that contain an explicit or implicit set of focus alternatives. Contexts that lack such a set do not reliably trigger the sentence type at hand. The authors conclude that the alternative semantics definition of focus (Rooth, 1985, 1992; Krifka, 2008) is also applicable to preVf.

Taken together, it has been proposed that preVf has the properties that have also been described by more general treatments of focus: it serves to identify, highlight, and in certain contexts, contrast information.

As stated earlier, the aims of the present work are 3-fold. The first aim is to gain further insight into the focus representation accounts. The secondary aim is to investigate the accessibility and representation of focused elements and its alternatives in the case of the Hungarian preVf. We investigated the issue in a WM task, and in a task that does not measure WM performance, such as a delayed recognition memory task. The tertiary aim pertains to the debate between the restrictive and permissive view of focus alternatives and amounts to investigating what can potentially belong to the alternative set evoked by preVf.

To investigate WM processes, we assessed immediate recognition memory performance in Experiment 1. This task required not only the storage but also the manipulation of WM representations; therefore, we refer to this paradigm as a Working Memory task instead of a short-term memory task (see e.g., Daneman and Carpenter, 1980). The aim of Experiment 2 was to assess the accessibility of memory representations with a delay before retrieval when participants are prevented from relying on WM processes. Since we did not aim to investigate long-term forgetting, following the tradition of experimental psychological research (see e.g., Tulving, 1985; and psycholinguistic research, see e.g., Spalek et al., 2014), we did not use a delay of days or even weeks between study and test, but a delay of a few minutes. During a delay participants are likely to keep repeating the verbal stimuli, and hence keep this information in WM (Cowan, 2008). To eliminate the possible effect of such rote rehearsal on memory performance (see e.g., McCabe, 2008), participants were asked to complete a non-interfering visual task with no memory component during this short delay.

## Experiment 1

### Predictions

Experiment 1 tested the potential effects of preVf on the accessibility and representation of focused elements and their alternatives using a probe recognition task in WM: participants were presented with stories in which we embedded a preVf containing sentence (PreVf sentence condition) or its focusless neutral counterpart (Neutral sentence condition). Immediately after each story, a probe sentence was presented to test the critical sentence. Three types of probes were used: (i) the probe was the same as the critical sentence in the story (Same probe condition), (ii) the focused word (or the corresponding word in the neutral sentence) was replaced by a semantically related word (Semantically related probe condition), or (iii) the focused word (or the corresponding word in the neutral sentence) was replaced by a semantically unrelated, but contextually suitable word (Contextually related probe condition). Conditions (ii) and (iii) will be collectively referred to as Different probe conditions.

The variables of interest were response latencies (which we will refer to as RT for reasons of convenience) and accuracy (i.e., rates of correct acceptance and rejection). In line with the probe recognition literature (see e.g., Sturt et al., 2004; Sanford et al., 2006; Jördens et al., 2020), these variables are taken as correlates of activation level. Also, RTs and correct rejection rates are a measure of relatedness in the Different probe conditions, since items which bear no semantic (or any other) relation to the critical item, i.e., those that are completely unrelated, are expected to be rejected highly accurately and fast compared to those which are in closer relation to the critical item. For this reason, results in RTs and correct rejection will show how the relatedness effect is modulated by activation level due to sentence type.

Regarding the Same probe conditions, we formulated our predictions as follows. Since all focus representation accounts claim that the representation of the focused element is enhanced relative to the non-focused elements, we predicted higher accuracy rates and lower RTs in the PreVf sentence condition relative to the Neutral sentence condition.

The Different probe conditions were introduced to test the predictions of the various focus representation accounts regarding alternatives. In terms of our dependent variables, the predictions derivable from the various accounts are formulated as follows. Regarding the semantically related alternatives, the granularity account suggests that alternatives of focused elements should be rejected faster and at a higher rate than items associated with non-focused elements. However, such patterns are not the consequence of a higher activation for focus alternatives, but are the result of a more fine grained representation of the focused element which even close associates are discriminated against. In contrast, the focus association account, whose point of departure is the claim that the function of focus is to indicate the presence of alternatives (see Krifka, 2008; Gotzner, 2017), explicitly predicts higher activation of focus alternatives. Thus, based on this account, we would expect to see faster reaction times and a higher rate of correct rejections of semantically related probes in the case of preVf sentences, than in the case of neutral sentences. Altogether, the predictions for the RTs and rejections of semantically related alternatives in the case of immediate recognition do not differ for the granularity and focus association theories, since these both suggest faster RTs and a higher correct rejection rate in the case of focus, albeit for different reasons. However, we will see that the predictions diverge for the delayed recognition experiment.

The Semantically related probe and the Contextually related probe conditions were introduced to test the predictions of the restrictive and permissive accounts of focus alternatives. As stated earlier, the restrictive view of focus alternatives suggests that focus activates semantic associates, while the permissive view claims that contextually suitable alternatives should also be activated even if they are not semantic associates. Along these lines, the restrictive view predicts that in the case of preVf, RTs should be faster and the rate of correct rejections should be higher only for the semantically related probes but not for the contextually suitable (but semantically unrelated) probes, since only semantically related focus alternatives should receive higher activation. On the contrary, the permissive view predicts that probes containing contextually suitable alternatives should also be responded to faster and should be rejected correctly at a higher rate in the case of preVf sentences. Thus, if the permissive view and the focus association account are correct, we expect to observe higher accuracy rates and faster responses in the PreVf sentence condition irrespective of the probe type.

The predictions will be tested using mixed effects models in which trial and participant will be included as random effects. The analysis will be carried out as outlined in Mirman (2014) and Bates et al. (2015).

### Method

#### Participants

Sample size for both Experiment 1 and 2 was determined based on previous work (e.g., Fraundorf et al., 2010; Gotzner et al., 2013). Forty undergraduate students recruited from the Budapest University of Technology and Economics participated in the experiment for course credit (27 females, *M*_*age*_ = 21.4 years, *SD* = 2.1). All participants in the experiments outlined in the present paper provided informed consent approved by the Hungarian United Ethical Review Committee for Research in Psychology. The study was carried out in accordance with the Code of Ethics of the World Medical Association (Declaration of Helsinki) for experiments involving humans. Participants in both experiments were native speakers of Hungarian and had normal vision or vision corrected-to-normal. Subjects had no history of psychiatric or neurological disorders.

#### Materials

The stimuli for the experiment were recorded in a sound treated room by a professional speaker. The speaker was asked to produce the linguistic material in a natural story-telling manner.

During the experiment, 36 experimental trials and 36 filler trials were presented auditorily. All trials contained a five-sentence story and a probe sentence with a 500 ms delay between the presentation of the story and the probe. An example of one experimental trial is provided in Table 1.

**Table 1:** Conditions and examples.

**Stimulus**	**Sentence- condition**	**Probe- condition**	**Example**
Story	PreVf: or Neutral:		A házibuli után Annára és Mikire hárult az elpakolás feladata. “After the party Ann and Mike undertook the work of tidying up.” Rendeztek mindent, ami a kezük ügyébe került. “They created order everywhere they went.” A konyhában is volt teendo elég. “There was a lot to do in the kitchen, as well.” Miki [egy tányért]_Focus_ rakott be a szekrénybe. “Mike put [a plate]_Focus_ in the cupboard.” Miki berakott [egy tányért] a szekrénybe. “Mike put [a plate] in the cupboard.” Aztán tovább sietett, és a bútorokat rendezgette. “Then he hurried on to arrange the furniture.”
Probe	PreVf	Same	Miki [egy tányért]_Focus_ rakott be a szekrénybe.
		Sem.-rel.	Miki [egy edényt]_Focus_ rakott be a szekrénybe (*plate* replaced with *pot*)
		Cont.-rel.	Miki [egy dobozt]_Focus_ rakott be a szekrénybe (*plate* replaced with *box*)
	Neutral	Same	Miki berakott [egy tányért] a szekrénybe.
		Sem.-rel.	Miki berakott [egy edényt] a szekrénybe (*plate* replaced with *pot*)
		Cont.-rel.	Miki berakott [egy dobozt] a szekrénybe (*plate* replaced with *box*)

*Critical NPs are in square brackets (Sem.-rel., Semantically related; Cont.-rel., Contextually related)*.

In experimental trials, the story contained either a preVf (PreVf sentence condition) or a neutral critical sentence (Neutral sentence condition). The critical sentences were either six or seven words long (*M*_*words*_ = 6.8, *SD* = 0.4), while the length of the stories varied between 39 and 43 words (*M*_*words*_ = 41.1, *SD* = 1.9). Each critical sentence contained a target word, which was the grammatical object of the sentence: an indefinite noun phrase (NP) in pre-verbal position in the PreVf sentence condition and in post-verbal position in the Neutral sentence condition. The critical sentence was the second, third or fourth sentence within the story. We varied the position of the critical sentence within the stories in order to eliminate potential confounds resulting from learning or practice effects: if the to-be-probed sentence was always in the same position within the stories, participants might develop an intuition about which sentence would be probed and might allocate extra attention to those sentences. The critical sentences were presented in the second, third, and fourth positions in an equal proportion of trials. Critical sentences never came first or last for two reasons. First, we wished to control for potential primacy and recency effects (Postman and Phillips, 1965). Second, it has been shown by Glenberg et al. (1987) that including a sentence between the critical sentence in the encoding phase and the probe sentence in the test phase allows sufficient time for a discourse representation to build up. Since in Experiment 1 probes immediately followed the stories, it was advisable to include at least one additional sentence intervening between the critical sentence and the probe.

Since sentences of two different information structure types (preVf and neutral) were presented in the same stories, the question arises whether these sentences differed in acceptability in their respective contexts. In order to ascertain that our results would not be confounded by different degrees of acceptability between the two sentence conditions, we conducted an online survey. In the trials of the survey, participants simultaneously read and heard the stories. In each story, the critical sentence was set in bold typeface. Participants rated the naturalness of these sentences using a 10-point Likert scale: value 1 corresponded to completely natural, while 10 corresponded to completely unnatural. Participants responded by clicking the numbers on the scale. We created two lists in order to eliminate the potential confounds resulting from presenting the same stories with both sentence types within one story: if the critical sentence was a preVf sentence in one story in one of the lists, this story contained its neutral counterpart in the other list. All 36 stories were presented together with 36 filler trials. In the filler trials the second, third, and fourth sentences were tested in an equal proportion, just as in the case of the test trials. Test and filler trials were presented in a randomized order. Thirty-nine university students took part in the survey (38 females, *M*_*age*_ = 20.7 years, *SD* = 1.2) for course credit. Participants were assigned to the lists randomly.

Results of the survey showed that the mean rating of preVf sentences was 3.028 (*SD* = 0.798), while the mean rating of neutral sentences was 3.027 (*SD* = 0.799). In order to test the hypothesis that the ratings of the two sentence types did not differ significantly, we built a Linear Mixed Effects Model using Sentence Type as fixed factor, and random intercept for Participant and Item. Models were built using the 1.1-21 version of the lme4 package (Bates et al., 2015). Running the model revealed that the variances in the data were close to zero (i.e., the model resulted in a singular fit), and therefore the model could not be built. In order to establish whether using a different distribution should lead to a better model, we used the fitdistrplus R package by Delignette-Muller and Dutang (2015) to estimate the distribution of our data. The analysis revealed a platykurtic distribution unsuitable for analysis by Mixed Models. For this reason, we resorted to using the Wilcoxon Signed-Rank Test (using the coin R package by Hothorn et al., 2006) which showed that the naturalness ratings of the two sentence types did not differ significantly (*Z* = 0.160, *p* = 0.873). Thus, we concluded that both sentence types fit the stories naturally, and potential confounds resulting from the use of unnatural linguistic stimuli could be eliminated.

Probe sentences were presented in three conditions: in the Same probe condition the probe was identical to the critical sentence; in the Semantically related probe condition the target NP was replaced by a semantically related word (e.g., *plate* replaced by *pot* for the example in Table 1); and in the Contextually related probe condition the target NP was replaced by a word that was contextually plausible but semantically unrelated to the target word (e.g., *plate* replaced by *box*). Probe sentences were recorded as whole. In other words, instead of splicing the critical words into the sentences, we made three recordings for each sentence type for the three conditions. This was done so that the prosodic characteristics of the preVf and neutral sentence type could be preserved, and thus the probe sentences sounded natural.

Using corpus data (the Hungarian National Corpus of 1.04 billion words, Oravecz et al., 2014), we matched the frequencies of words used as target in the critical and probe sentences: a comparison revealed that there was no significant difference between the words in the three conditions (Same, Semantically related, and Contextually related probe conditions), *F*_(2, 107)_ = 0.705, *p* = 0.496. Also, the lengths of these words were controlled: we used nouns with lengths of two or three syllables in their accusative case. Within trials, word forms of the same number of syllables were used.

The structure of the 36 filler trials was identical to those of the critical trials: each contained a five-sentence story and a probe. Half of the probes were identical to one of the sentences in the story while the other half contained a change. The position of the probed sentence within the stories was also balanced in the fillers. None of the filler sentences had a preVf structure, and no replacements in filler probes involved the object NP.

#### Procedure

The experiments presented in the current paper were programmed with Matlab R2014a using the Psychtoolbox version 3.0 (Brainard, 1997; Pelli, 1997; Kleiner et al., 2007; MATLAB, 2010).

After filling in the consent form, participants were seated in front of a computer screen with headphones on and were given instructions. They were informed that the probes would occasionally contain some change, therefore they were requested to pay special attention to all aspects of the stories and give their response as accurately and as fast as possible. Thus, the encoding was intentional, as subjects were required to memorize the stories. Participants responded by button presses corresponding to the following instruction: If the sentence you just heard is identical to any of the sentences in the previous story, press “yes,” if you detect any change, press “no.” Practice trials were not included, since as a consequence of the block structure (i.e., all blocks started with a filler) no experiment started with a critical trial. One trial was sufficient for participants to understand the experimental task.

Each item appeared in only one condition for each participant. The structure of one trial was as follows: a fixation cross appeared on the screen, and the story was presented auditorily. The fixation cross appeared at the onset of the story and remained on the screen until its end. Following the story, a black question mark appeared in the place of the fixation cross and the probe sentence was presented. Both the presentation of the story and the probe sentence were preceded by a 500-ms inter-stimulus interval. When the probe sentence ended, the question mark turned green and the participant could press the button corresponding to their response. Participants were encouraged to respond as quickly and as accurately as they could. Maximally 8 s were allowed for responses to be made. Trials were presented in six blocks, and each block contained 12 trials. The allocation of trials to the blocks was randomized, as well as their order within the blocks with one constraint: the first and last stories of each block were fillers. Between the blocks, participants played a visual game on a tablet for 2 min to eliminate the possible effect of rote rehearsal on memory and to circumvent fatigue effects. The average duration of a recording session was 60 min.

### Results

All analyses presented in the current paper were carried out in R version 3.5.3 using the 1.1-21 version of the lme4 package (Bates et al., 2015; R Core Team, 2019). Data obtained in the Same and Different probe conditions were analyzed separately for reasons outlined in the Predictions section.

Looking at the Same probe condition, response accuracies were fairly high in both sentence conditions (*M* = 82.9%, *SD* = 16.7 for neutral sentences, *M* = 86.60%, *SD* = 15.2 for preVf sentences). In order to test our predictions regarding this measure in the Same probe condition, we built generalized linear mixed effect models to predict Accuracy using the binomial distribution in successive steps (Bates et al., 2015). First, a base model was built with an Intercept and then a model with Sentence Type as predictor. Random effects included random intercept for trial and random intercept for participant. A likelihood ratio test comparing the two models did not show an improvement in fit [χ${\;}_{\left(1\right)}^{2}$ = 1.126, *p* = 0.289]. Thus, contrary to our expectations, Accuracy in the Same probe condition did not differ significantly between the two sentence types.

**Figure 1:**
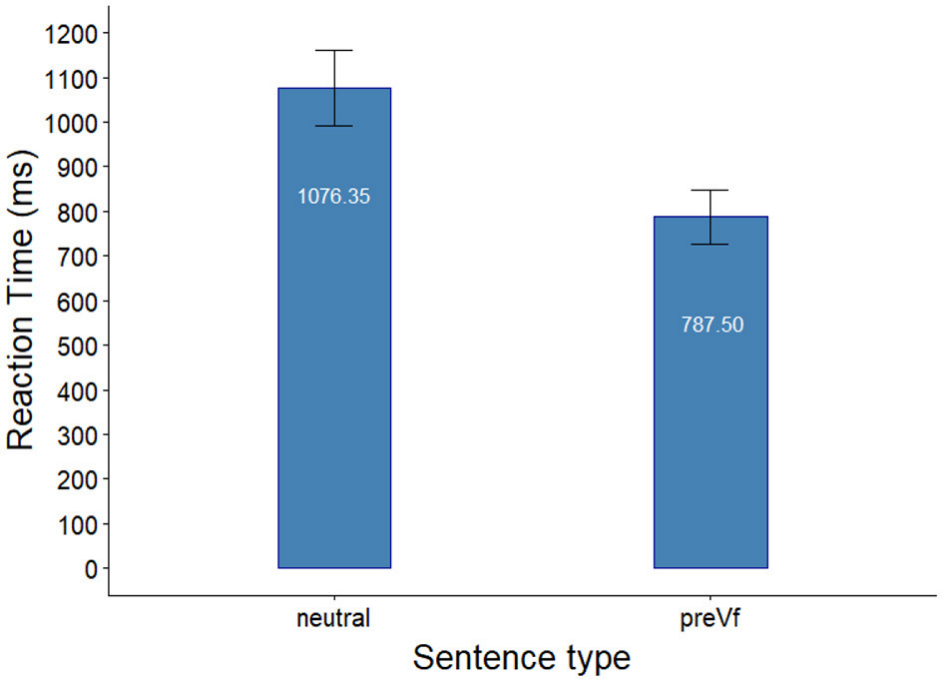
RTs in the Same-conditions in Experiment 1. Error bars indicate the standard error of the mean.

RT data from the Same probe condition is presented in Figure 1.

As a next step, we analyzed RT data obtained in the Same probe condition. Trials in which participants gave incorrect responses were excluded from the analysis of RT (15.3%). We fitted mixed effects regression models to the data in two successive steps. First, an intercept-only base model was built, second, Sentence Type was added as predictor. The random effect structure for the models was random intercept for Participant and random intercept for Trial. Addition of further random effects resulted in non-convergence of the models. The comparison of the two models was carried out using the likelihood ratio test, which showed a significant improvement in fit by the addition of Sentence Type [χ${\;}_{\left(1\right)}^{2}$ = 9.388, *p* = 0.002] revealing that participants responded faster in the PreVf sentence condition than in the Neutral sentence condition. The model including the predictor Sentence Type and its parameter estimates are presented in Table 2.

**Table 2:** The best fitting model and its parameter estimates predicting RT in the same condition in Experiment 1.

RT ~ sentence_type + (1 | Participant) + (1 | Trial)					
	**Estimate**	**Std. Error**	***df***	***t***	***p***
Intercept	1087.631	98.040	74.078	11.094	0.000
Sentence type_ preVf	−293.640	95.049	359.749	−3.089	0.002

**Figure 2:**
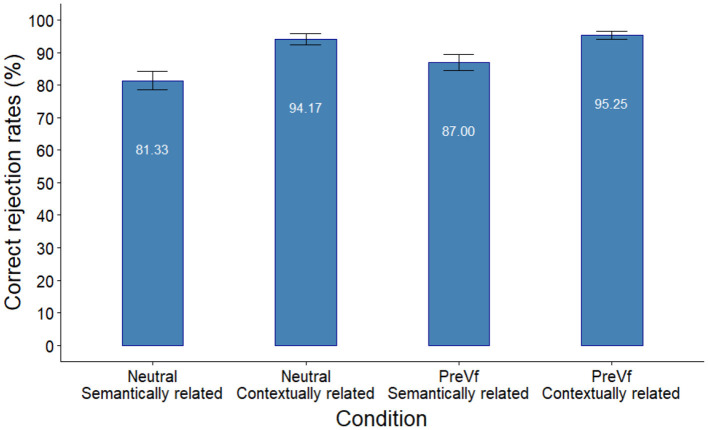
Accuracy rates in the different probe conditions in Experiment 1. Error bars indicate the standard error of the mean.

Accuracy rates in the Different probe conditions are presented in Figure 2.

Accuracy in the Different probe conditions was analyzed using logistic mixed effects models with binomial distribution. The predictors Sentence type and Probe type were contrast coded using the effects R package by Fox and Sanford (2019). The random effect structure was random intercept for participant and random intercept for trial. Models were built in successive steps by adding fixed effects to an intercept only base model. Addition of Probe Type resulted in a better fit [χ${\;}_{\left(1\right)}^{2}$ = 30.827, *p* < 0.001] showing better performance in the case of contextually related probes, while the inclusion of Sentence Type missed the level of significance [χ${\;}_{\left(1\right)}^{2}$ = 3.499, *p* = 0.061] in the improvement of model fit. Adding the interaction term Sentence Type × Probe Type did not improve fit [χ${\;}_{\left(1\right)}^{2}$ = 0.389, *p* = 0.533). The specification and parameter estimates of the best fitting model are presented in Table 3.

**Table 3:** The specification and parameter estimates of the best fitting model predicting accuracy in the different probe conditions in Experiment 1.

accuracy ~ probe_type + sentence_type + (1 | Participant) + (1 | Trial), family = binomial				
	**Estimate**	**Std. Error**	***z***	***p***
Intercept	2.547	0.209	12.171	<0.001
Probe type	0.660	0.126	5.225	<0.001
Sentence type	−0.212	0.114	−1.856	0.063

Finally, we analyzed the RTs of correct rejections in the Different probe conditions [PreVf—semantically related: *M*_*RT*_ = 893.535 (1118.442), contextually related: *M*_*RT*_ = 555.387 (565.020); Neutral—semantically related: *M*_*RT*_ = 912.105 (1002.323), contextually related: *M*_*RT*_ = 683.562 (789.095)]. We included Probe Type and Sentence Type as contrast coded predictors in the statistical analysis. The random effect structure of the models was random intercept for participant and random intercept for trial. First, a base model was built and the two predictors were added in two successive steps. Models were compared using the likelihood ratio test. Addition of Probe Type resulted in a better fit [χ${\;}_{\left(1\right)}^{2}$ = 11.394, *p* < 0.001], showing faster RTs for the contextually related probes. However, the inclusion of Sentence Type did not result in a better fit [χ${\;}_{\left(1\right)}^{2}$ = 1.982, *p* = 0.159] indicating that sentence type did not have an effect on RTs.

### Discussion

Experiment 1 investigated the memory accessibility and representation of the focused element and its alternatives in the case of preVf in WM. Regarding the accessibility of the focused element, accuracy (i.e., correct responses on the immediate recognition memory test) did not indicate an advantage. However, RT did show that preVf had a facilitative effect. Despite the lack of advantage in the case of accuracy, we conclude that RT alone shows the facilitatory effect of focus on the accessibility of the critical element, since this measure is a correlate of the durations of processes (or stages) that take place when the correct recognition of an item occurs (Sternberg, 1969). The discrepancy between accuracy and RT may be attributable to the difference in the sensitivity of these measures of memory accessibility. Furthermore, the reason we did not find an effect in terms of detection rates found by for example Sturt et al. (2004) and Sanford et al. (2006) may be a methodological one: while these authors used three-sentence texts, we used five-sentence stories which may have made the experimental task used in our investigation more difficult. Since, as mentioned above, accuracy is a less sensitive measure regarding accessibility than RT, accuracy did not show a difference in this more difficult task. This assumption needs to be addressed in later work.

Regarding the different conditions, it has been found that relatedness has an effect: overall, contextually suitable but semantically unrelated alternatives were better recognized than semantically related alternatives. This is an expected result, since the difference between semantically unrelated items is more salient irrespective of context and sentence type. No statistically reliable difference has been found, however, between the two sentence types in the rejection rates of different probes, i.e., the data provide no support for the focus association account, which claims that focus leads to the relatively higher activation of alternatives. Nevertheless, the close to significant effect indicates that this activation may be higher which may have remained undetected due to methodological reasons. This suggestion will be addressed in the General Discussion section, and it will be shown that there is indirect evidence for the higher activation of focus alternatives compared to non-focused ones. Since the effect of preVf on the activation of alternatives was not detected in Experiment 1, our results are also inconclusive regarding the restrictive versus the permissive accounts of focus alternatives. In future work, methodological improvement is needed to address this question.

As far as RT is concerned, we found an effect of probe type: participants responded to probes containing a semantically unrelated but contextually suitable alternative faster than to probes with semantically related alternatives. We believe that the observed difference in RT is also attributable to the relatedness effect discussed above. However, contrary to our predictions, sentence type did not have an effect, that is, participants responded to both probe types similarly fast.

## Experiment 2

The purpose of Experiment 2 was to test the predictions of the different focus representation accounts regarding delayed recognition, i.e., when an individual is not able to rely on WM processes for maintaining information. For this reason, the stories were presented in blocks, and probes were presented after the stories with a delay (for a similar method, see Spalek et al., 2014).

### Predictions

As in the case of Experiment 1, we made predictions regarding accuracy rates and RTs. As far as the accessibility of the focused element is concerned (i.e., Same probe condition), the granularity account and the contrast account both predict an enhancement in the accessibility of the focused element. Therefore, if either one of these theories is tenable, we should see an advantage of preVf sentences which should be manifest in higher accuracy rates and lower RTs relative to the Neutral sentence condition. Additionally, the focus association hypothesis makes no specific prediction but when testing this account, Spalek et al. (2014) found no effect for the German particles *nur* (only) and *sogar* (even).

Regarding the accuracy rates in the Different probe conditions, the granularity account does not make a prediction regarding the accessibility of focus alternatives. On the other hand, the contrast account predicts an enhancement in the accessibility of mentioned contrastive alternatives, but not for unmentioned alternatives (Fraundorf et al., 2010). Since we did not use mentioned alternatives in our context stories, the contrast account predicts no effect of focus on the correct rejection of the different probes. At the same time, the focus association account predicts that interference should occur: since focus alternatives (which can be either mentioned or unmentioned) receive a higher activation in WM, we should see an interference after a delay. Such an effect is expected, as representations of similar semantic content have been shown to interfere (Baddeley and Dale, 1966; see also Baddeley, 2009; Baddeley et al., 2020) when there is a delay before the retrieval of memory elements. In terms of our dependent variables, this translates as higher RT and a lower correct rejection rate in the PreVf sentence condition relative to the Neutral sentence condition for the different probes.

Just as in the case of Experiment 1, the predictions will be tested using mixed effects models in which trial and participant will be included as random effects. The analysis will be carried out as outlined in Mirman (2014) and Bates et al. (2015).

### Method

#### Participants

Forty undergraduate students recruited from the Budapest University of Technology and Economics participated in the experiment for course credit (34 female, *M*_*age*_ = 23.0, *SD* = 1.8).

#### Materials

The stimulus set used in Experiment 2 was identical to the one used in Experiment 1.

#### Procedure

The experimental design and the procedure were identical in Experiments 1 and 2 with only one crucial modification. While the presentation of a story was always immediately followed by the presentation of a probe in Experiment 1, probes were presented at the end of each block in Experiment 2. Each item only appeared in one condition. The experiment consisted of 12 blocks, each containing six trials. The allocation of trials to the blocks was randomized, as well as their order within the blocks. The structure of the blocks in Experiment 2 was as follows: first, a set of six stories (with 500 ms delays between each) was presented followed by a 2-min delay. During the 2-min delay participants played a visual game on a tablet in order to eliminate the effect of rote rehearsal on memory retention. After the game, participants returned to the computer and were presented a series of probes. The order of the probes corresponding to the stories was identical to the order of the stories. As in Experiment 1, participants saw a black question mark during the probe. When the probe sentence ended, the question mark turned green, and the participant could give their response. The experimental task was the same as in Experiment 1: participants were asked to respond by button presses corresponding to the following instruction: If the sentence you just heard is identical to any of the sentences in any of the stories you heard in the previous set of stories, press “yes,” if you detect any change, press “no.” Participants were allowed a maximum of 8 s to respond. The duration of one experimental session was ~60 min.

### Results

As in the case of Experiment 1, data obtained in the Same and Different probe conditions were analyzed separately.

First, we carried out a statistical analysis of the accuracy rates in the Same probe condition. The same procedure was followed as in the case of Experiment 1: a base model with Intercept and a model with Sentence Type as predictor was built. Comparison of the two models did not reveal an improvement in fit [χ${\;}_{\left(1\right)}^{2}$ = 3.075, *p* = 0.08], showing that Sentence Type had no effect on Accuracy in the same-condition. Note, however, that the difference between the two sentence types suggests a tendency in the opposite of the predicted direction: 63.33% (*SD* = 20.74) for preVf sentences and 71.25% (*SD* = 23.56) for neutral sentences.

**Figure 3:**
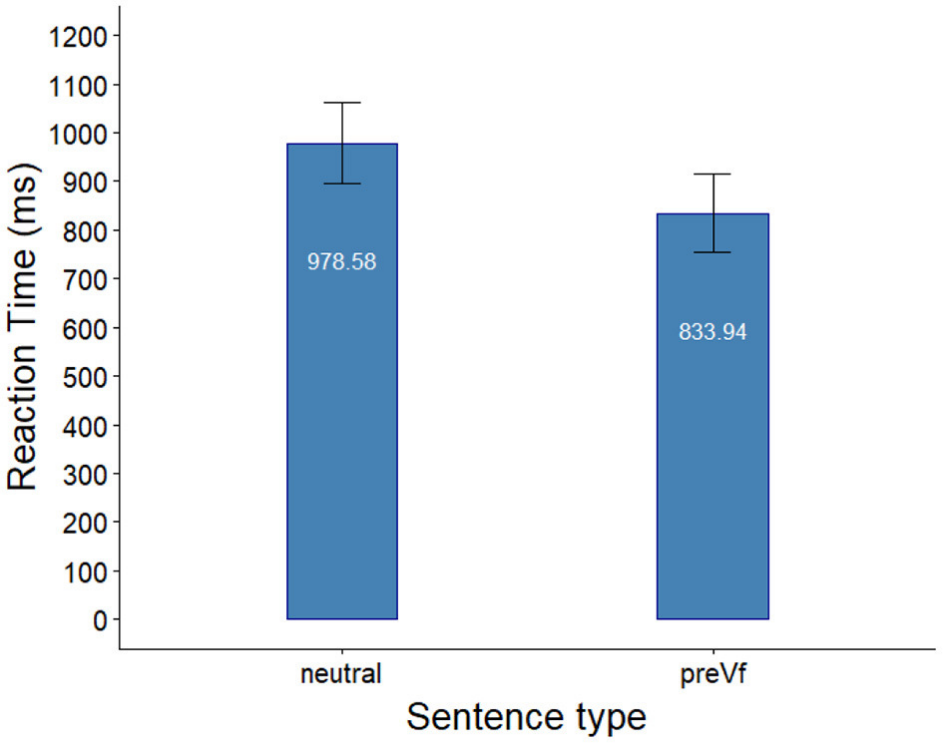
Response latencies in the same-condition in Experiment 2. Error bars indicate the standard error of the mean.

After the exclusion of trials in which participants gave incorrect responses (32.71 %), RT data from the Same probe condition were analyzed using mixed effects models with random intercept for Participant and random intercept for Trial as random effects. First, a base model with Intercept as predictor was built to which we added Sentence Type as predictor. The likelihood ratio test showed no significant improvement in model fit for sentence type [χ${\;}_{\left(1\right)}^{2}$ = 1.678, *p* = 0.195] meaning that response latencies for the two sentence types did not differ significantly. RT data are presented in Figure 3.

**Figure 4:**
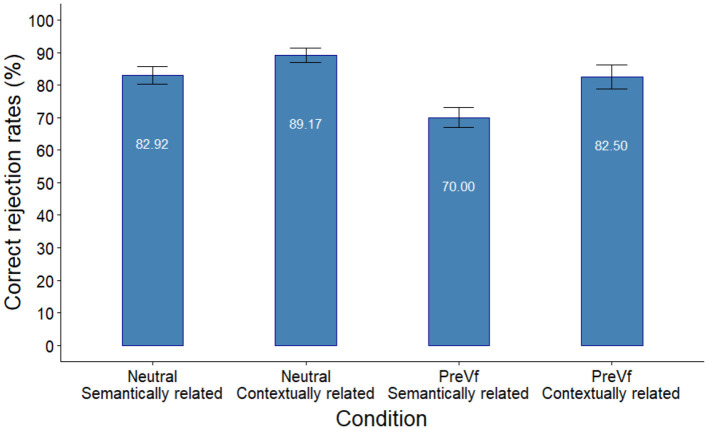
Accuracy rates in the different probe conditions in Experiment 2. Error bars indicate the standard error of the mean.

Accuracy data obtained in the Different probe conditions are presented in Figure 4.

We built logistic mixed effects models using binomial distribution to analyze Accuracy in the Different probe conditions. The predictors Sentence type and Probe type were contrast coded using the effects R package by Fox and Sanford (2019). Random factors included random intercept for Participant and random intercept for Trial. Addition of further random factors led to non-convergence. Models were successively built by adding fixed effects to an intercept only base model. Addition of Probe Type resulted in a better fit [χ${\;}_{\left(1\right)}^{2}$ = 15.266, *p* < 0.001] showing worse performance in the case of semantically related probes, and crucially, so did addition of Sentence Type [χ${\;}_{\left(1\right)}^{2}$ = 6.794, *p* = 0.01] indicating that participants' memory performance was worse in the PreVf sentence condition. However, inclusion of the Probe Type × Sentence Type interaction term did not improve fit [χ${\;}_{\left(1\right)}^{2}$ = 0.134, *p* = 0.714]. The specification and parameter estimates of the best fitting model are presented in Table 4.

**Table 4:** The specification and parameter estimates of the best fitting model predicting accuracy in the Different probe conditions in Experiment 2.

accuracy ~ probe_type + sentence_type + (1 | Participant) + (1 | Trial), family = binomial				
	**Estimate**	**Std. Error**	***z***	***p***
Intercept	1.858	0.209	8.887	<0.001
Probe type	0.360	0.091	3.932	<0.001
Sentence type	0.399	0.147	2.707	0.007

As a final step, we analyzed RTs of the correct rejections in the Different probe conditions [PreVf—semantically related: *M*_*RT*_ = 997.130 (1147.003), contextually related: *M*_*RT*_ = 975.848 (1194.569); Neutral—semantically related: *M*_*RT*_ = 879.031 (916.145), contextually related: *M*_*RT*_ = 916.259 (923.020)]. In the statistical analysis, we included Probe Type and Sentence Type as contrast coded predictors. The random effect structure of the models was random intercept for participant and random intercept for trial. First, a base model was built and the two predictors were added in two successive steps. Models were compared using the likelihood ratio test. Addition of Probe Type did not result in a better fit [χ${\;}_{\left(1\right)}^{2}$ = 0.078, *p* = 0.779], and neither did the inclusion of Sentence Type [χ${\;}_{\left(1\right)}^{2}$ = 0.024, *p* = 0.878] indicating that none of the two predictors had an effect on RTs.

### Discussion

Experiment 2 investigated the memory accessibility and representation of the focused element and its alternatives in the case of preVf as measured by response accuracy and RTs of correct responses on a delayed recognition memory test. In delayed recognition memory tests we assume that participants are not able to rely exclusively on processes for maintaining information in WM. Regarding the focused element, we found a tendency toward a difference in accuracy rates opposite of what we predicted: memory performance for focus seems to be worse than for neutral counterparts in delayed recognition. In the current framework this result is highly surprising, and it is very difficult to offer a non-speculative explanation, especially in the light of results on RT, which suggest that preVf may not have a facilitative effect in a delayed recognition memory task. Note again, however, that there may be some degree of ambiguity in the results, as the experiment contained only six trials in the given condition leading to an insufficient level of statistical power. Nevertheless, the results on accuracy and RT jointly suggest that the lack of facilitative effect in delayed recognition. Indeed, this finding is in line with the results of Spalek et al. (2014), which demonstrated that German focus particles (*nur* and *sogar*) had no facilitative effect on the recall of the focused elements themselves. A potential explanation as to why focus may indeed not have a facilitative effect will be offered in the General Discussion section. With respect to the alternatives (i.e., Different probe condition), the results show that the accuracy of rejections was overall lower for the preVf sentences than for the neutral sentences. This overall effect is a consequence of semantic interference (Baddeley, 1966, 2009; Baddeley et al., 2020) and is in line with the predictions of the focus association account: Since the function of focus is to mark the presence of alternatives, not only the focused element but also its alternatives are activated. Following a delay, these activated elements interfere with each other, which is reflected in the deterioration of memory performance for these items. Also, as in Experiment 1, overall accuracy for semantically unrelated but contextually related alternatives was better than for semantically related alternatives. Just as previously, this was an expected result since the difference between semantically unrelated items is more salient than between semantically related ones. Regarding the restrictive and permissive views of focus alternatives, the lack of interaction does not enable us to adjudicate between the two accounts. Just as in the case of Experiment 1, further refinement of methodology is needed to address the question of what constitutes the set of alternatives in the case of the Hungarian preVf.

Similarly to Experiment 1, none of our predictions regarding RTs have been confirmed: we found no effect of probe type and no effect of sentence type. These results will be discussed jointly with the ones from Experiment 1 in the General Discussion section below.

## General Discussion

The current study investigated the representation and the accessibility of focused elements and their alternatives in the case of Hungarian pre-verbal focus (preVf) in two probe recognition experiments with no delay and with a two-minute delay between encoding and retrieval. The study investigated three main questions related to focus representation in WM, and the accessibility to these representations when one is not able to rely on processes for maintaining information in WM: (i) the accessibility of the focused element, (ii) the activation of the focus alternatives, and (iii) the question of what constitutes the set of focus alternatives. In the following, our findings pertaining to these questions will be discussed.

First, however, a note regarding the use of the preVf construction is due with relation to our research questions. As outlined earlier, focus in preVf is marked by two features: (i) the inverted configuration of the verbal modifier (if there is one) and the verb with the focused element sitting immediately pre-verbally (a syntactic feature), and (ii) the eradicating stress on the focused element (a prosodic feature). One could consider the objection that observing any memory-related effect in the case of this construction introduces an indeterminacy regarding what can be inferred from the data: perhaps, prosody alone would produce the observed effects. However, this objection is hardly tenable, since the Hungarian structural focus is jointly characterized by the immediately pre-verbal position and the eradicating stress assigned to it, even if a verbal modifier is absent from the sentence. In other words, the focus type at hand has two central defining features and any study separately investigating the potential effects of these features would be questionable regarding its content validity. Furthermore, such an objection would render findings inconclusive also on clefts, since focus is also doubly marked in this type of construction, i.e., by syntax and prosody. Finally, since no work has been carried out addressing the issue of memory accessibility and representation of Hungarian focus so far, we decided to start investigating the construction that is the most representative example of Hungarian focus; namely, the pre-verbal focus as presented in (3a).

With respect to the memory accessibility of the focused element in WM, our results are in line with findings in the international literature: response latencies revealed that the focused element is more readily accessible in memory. This finding supports the traditional theoretical and functional definitions (see e.g., von Stechow, 1991; Krifka, 1992) according to which focus highlights or foregrounds information, or, as Sanford et al. (2006) phrased it, focus functions as an attention capturing device.

We believe that this psychological effect is utilized in organizing discourse in a coherent and efficient way. For example, the relatively higher accessibility of focused elements may explain the observations made in the theoretical literature regarding “focused topics.” Such phenomena are called continuous-topic constructions, and have been observed in English (Prince, 1978; den Dikken, 2013), German (Huber, 2004), and also in Hungarian (Gécseg, 2020). An example by Kayne (2014, p. 195) is given in (4).

(4) A: Do you know Mary?
B: Yes, in fact it was [Mary] who/that I learned linguistics from in the first place.

Note, that the clefted, i.e., syntactically focused element in B's answer in (4) functions as topic: it encodes an entity that has been introduced into the discourse, and also this entity is the one about which the wh-clause makes a statement. We posit that the reason why such so-called aboutness topics tend to be focused syntactically is that in this way they become more accessible in memory while discourse about the topic unfolds. This mechanism is thus key in efficiently managing discourse by locally enhancing the representation of the entity or entities that are central during an act of communication. Note also, that we found no advantage for the focused element after a delay which also suggests that this property of focus is used for relatively local purposes, such as discourse organization. This consideration is also supported by brain imaging results which have shown that the processing of focus containing sentences activates areas implicated in discourse processing (see e.g., Spalek and Oganian, 2019).

As far as the focus alternatives are concerned, Experiment 1 found no direct evidence for an increased activation in WM as measured by immediate recognition memory performance. However, it must be pointed out that the effect was close to significant, and that the observed accuracy rates showed a tendency in the predicted direction. One might argue that these results suggest that a higher activation of alternatives takes place, while the lack of significant results is a consequence of an insufficient amount of data. Note, however, the pattern of results obtained in the Different conditions in Experiment 2, may serve as independent evidence for the claim that focus *indeed* activates alternatives (just as the close to significant effect in Experiment 1 might also be indicative of this higher activation). We conjecture that the pattern of results in Experiment 2 is the consequence of semantic interference. The interference observed in the case of both probe types was most probably the result of a higher activation of both semantically related and contextually suitable alternatives upon the processing of the focus containing sentences. For this reason, we conclude that the results on the correct rejection rates obtained in the two experiments jointly corroborate the psychological reality of theoretical accounts capturing focusation in terms of evoking alternatives (Rooth, 1992; Krifka, 2008). As far as the restrictive and permissive views on focus alternatives are concerned, however, our results are inconclusive. Thus, further research and a refinement of methodology is needed in order to adjudicate between the two views on what constitutes the set of alternatives.

As far as response latencies of correct rejections in the WM task are concerned, we found that probes with semantically unrelated but contextually suitable alternatives were responded to faster than probes with semantically related alternatives. Additionally, no effect on response latencies was found for correct rejections in the delayed recognition memory task. It is highly likely that the effect of probe type in WM is associated with relatedness: since contextually related probes contained a semantically unrelated alternative, this probe type was more easily discriminable, which led to faster rejections. We speculate that the reason for why this effect was not observed in the delayed recognition memory is that our measurement was not sensitive enough for the measurement of such effects. Nevertheless, it is much more likely that there is no reliable link between response latencies associated with correct rejections and the activation strength of critical elements, as a number of different processes may be operative during a correct rejection in the tasks used in our experiments. These processes may for example be familiarity-based decisions or “recall-to-reject” processes. Therefore, response latencies may reflect different processes in different trials and different participants within an experiment, making this measure unreliable. This might also be the reason for why we found no effect of sentence type on RT as opposed to accuracies of correct rejections. The investigation of these possibilities requires further experimentation.

Before turning our attention to other aspects of our findings, let us discuss an alternative explanation of results also mentioned in section Discussion. According to this interpretation, the observed benefit of preVf on the correct rejection of alternatives in WM may have been the result of novelty instead of the generation of alternatives: perhaps, due to the attention controlling properties of focus, the focused elements had a higher activation, and consequently, the rejection of any element sitting in the focus position of the probe sentence may have been easier. If this was indeed the case, it is difficult to clearly understand why a worse performance was observed in the case of both probe types in the PreVf sentence condition in Experiment 2: only those elements can interfere that gain some level of activation.

Regarding the accessibility of focused elements in Experiment 2, as measured by delayed recognition memory performance (i.e., when one is not able to rely on WM processes), we found no reliable results. Accuracy results suggest a tendency in the opposite direction of what we predicted: memory performance seems to be worse for focus than for neutral sentences. However, we found no evidence for this effect in the RT data. As mentioned earlier, these results were not predicted by any of the focus representation accounts: while Fraundorf et al. (2010) found a facilitative effect on the accessibility to prosodically focused elements, Birch and Garnsey (1995) and Almor and Eimas (2008) found an adverse effect for syntactically focused elements. There was, however, one study which showed that elements marked by the German focus particles *nur* (*only*) and *sogar* (*even*) were not retrieved at a higher rate than elements without these particles (Spalek et al., 2014). Since our results are inconclusive regarding the accessibility of focused element in delayed recognitions, the question should be investigated in future studies.

One explanation for why there indeed may be a lack of enhancement in Experiment 2 is that the gist of the sentences is retained in memory for longer periods of time, while their exact syntactic realization is lost (see e.g., Sachs, 1967; Johnson-Laird et al., 1970; Samuel, 1972; Flores D'Arcais, 1974; Graesser and Mandler, 1975; Gernsbacher, 1985; Anderson et al., 2001). In the above studies, gist is defined as the semantic representation of the sentence as opposed to the representation of its surface form, or more specifically, a representation which may, for instance, eliminate the distinction between an active and a passive sentence. In other words, gist is nothing more than the core meaning of a sentence (Anderson et al., 2001). In the case of preVf and neutral sentences used in our experiments, it is reasonable to assume that the gist of these sentences was equivalent to the relation that they expressed between the subject and object determined or modified by the adverb. For example, in the case of the sentence *Miki [egy tányért]*_*Foc*_ *rakott be a szekrénybe* (~ *Mike put [a plate]*_*Foc*_ *in the cupboard*), the gist is the relationship between the plate and Mike such that the former was put into the cupboard by the latter. However, one might raise the objection that exhaustivity is also part of the core semantic meaning of preVf sentences, as opposed to neutral sentences, in which exhaustivity is not assumed to be represented semantically. Such theories, however (see e.g., Kiss, 1998; Kenesei, 2006) have not been supported by experimental data (see e.g., Onea and Beaver, 2009; Kas and Lukács, 2013; Gerocs et al., 2014; Káldi et al., 2016; Káldi and Babarczy, 2018). Furthermore, recent experimental evidence suggest that the use of focus may not only be strictly motivated by linguistic factors, and that these factors should be best seen as pragmatic ones (Stevens and Roberts, 2019; Káldi et al., 2020). Therefore, we contend that the gist of the two sentence types at hand were the same in our experiments. Their apparent syntactic and prosodic differences belong to their surface characteristics.

The explanation relying on the assumption that the gist is retained as opposed to the form for longer periods of time becomes especially plausible, if we consider one of the central functional properties of focusation: namely, focus serves to organize discourse, partly by introducing new referents. Consider the dialogue in (5) in which A's question requests the identification of the individuals invited by John. The answer in B1 is acceptable, since the element carrying new information is marked for focus, while the answer in B2 sounds rather odd, since the respective element sits post-verbally (for further theoretical explanation see Roberts, 1998; Surányi, 2011, for experimental results see Káldi et al., 2020).

(5) A: Kit hívott meg János?
  Who did John invite?
B1: János Marit hívta meg (preVf)
  John invited ‘Mary.
B2: #János meghívta Marit (neutral)
  John invited Mary.

Note, however, that the “gist” of the two answers in (5) is the same: there is a relation between John and Mary such that the former invited the latter. The information structural properties of the sentence realized in a particular syntactic construction in the case of preVf serves local discourse purposes. For this reason, the syntactic form of the sentence may lose its relevance for longer periods of time and is not retained in memory. The gist, however, is retained irrespective of the syntactic structure as revealed by the relatively high recognition rates. We believe that the above considerations may open up a new line of research studying the interrelation of syntactic structure and information structure in memory (for one such study see Pléh and Sinkovics, 2011). As noted earlier, our results are suggestive of, but inconclusive regarding the longer effects of focus on memory representation. Hence, we propose that the above outlined explanations should be considered as a basis for further research on the long term accessibility to focused elements.

With respect to both semantically and contextually related focus alternatives in the delayed recognition memory task, we found that memory performance for these items was poorer than for alternatives to non-focused counterparts. On the face of it, two explanations offer themselves for the observed results. One of these has already been mentioned: the poorer recognition rates may be attributable to semantic interference generally observed when there is a delay before retrieval (e.g., Baddeley, 1966, 2009; Baddeley et al., 2020): focus activates the representation of alternatives (as also suggested by the results of Experiment 1), and these highly activated semantic representations interfere with each other. The interference leads to low recognition rates. Alternatively, since the experimental task was to decide if the probe was identical to the target (i.e., old) or it was different (i.e., new) we can say that the observed poorer performance is not the result of a lower tendency to correctly recognize the critical element, but it is the result of a greater tendency to falsely recognize it. The effect of creating false memories at the level of associated items has been extensively studied and repeatedly replicated in the memory literature (Deese, 1959; Underwood, 1965; Anisfeld and Knapp, 1968; Hintzman, 1988). For example, Roediger and McDermott (1995) presented lists of associated words to their participants which had to be recalled or recognized after a 5-min delay. The main finding of the study relevant to our purposes was that recognition memory was affected by the semantic association between list items: the proportions of hit rates and false alarm rates were identical suggesting that participants had not been able to distinguish between actually presented items and items that had not been presented in the lists. According to Roediger and McDermott (1995, p. 810) the effect is “produced by means of activation of implicit associative responses.” Thus, one may raise the objection that the effect observed in the Different probe condition of Experiment 2 is at least partly attributable to this activation mechanism. However, it is hard to see how focus could modulate this mechanism without assuming that it indeed activates alternatives. To conclude, the most plausible explanation is that the lower rate of correct rejections in the case of preVf sentences was the result of a greater semantic interference of activated alternatives. This explanation is also in line with other findings in the literature (see e.g., Spalek et al., 2014; Gotzner, 2017).

In sum, the present work investigated the memory accessibility of linguistically focused elements and their representation in WM and when one is not able to rely on processes for maintaining information in WM in the case of the Hungarian pre-verbal focus construction. It has been shown that focus enhances the accessibility of the focused element in an immediate recognition memory task and most probably it has no facilitating effect on a delayed recognition memory test indicating a dissociation between WM and delayed recognition memory performance. While the former effect can be explained by the attention capturing property of focus (Brassai, 1860; Sanford et al., 2006), the latter observation may be attributable to the tendency that gist is retained longer than form. Furthermore, we have provided indirect evidence that preVf evokes the representation of a set of alternatives. This indirect evidence comes from the finding that the memory performance for focus alternatives is poorer for longer periods of time: this effect is most probably the result of semantic interference, for which the best explanation is that focus does activate alternatives in WM.

Finally, let us discuss two potential methodological limitations of our study. One of the limitations concerns tendencies toward a difference regarding sentence type in the Different-conditions in Experiment 1, and in the Same-condition in Experiment 2. As pointed out earlier, these almost significant results may have been the consequence of an insufficient amount of data, as the number of trials in the conditions was rather low. It is highly likely that this has lent some ambiguity to our results. Reducing the number of conditions would enable the future researcher to increase the trials in one condition without dramatically increasing the length of the experiment. The other potential limitation concerns RT measures. Note that while earlier studies, such as Fraundorf et al. (2010), Gotzner and Spalek (2016), etc., used words as probes, our experiments used sentences. This may have led to a substantial variability in the measured RT values which also makes it difficult to formulate solid conclusions regarding our research questions. However, we firmly believe that the results presented here are valuable for both the psycholinguistic theories of focus in general and for the Hungarian focus in particular, and that the limitations outlined above will motivate further research on the issues at hand.

As far as the methodological novelty is concerned, since both experiments used exactly the same stimulus set (auditorily presented stories followed by probe sentences), and they only differed in terms of the timing of recognition probes, the principle of ceteris paribus fully applied with respect to how we addressed our research question regarding the two different memory processes. Thus, to our knowledge, this is the first study that investigates the focus representation accounts for WM and delayed recognition of focus alternatives in this principled manner. Also, no study has been conducted on the memory representation of focused elements and their alternatives in the case of the Hungarian pre-verbal focus construction.

## Data Availability Statement

The datasets generated for this study are available on request to the corresponding author.

## Ethics Statement

The studies involving human participants were reviewed and approved by Hungarian United Ethical Review Committee for Research in Psychology. The patients/participants provided their written informed consent to participate in this study.

## Author Contributions

TK conceived of the presented idea. The experimental materials were created and recorded by TK and ÁS. Data analysis was carried out by TK with the supervision of AB. The manuscript was written by TK with support of AB and ÁS. All authors were involved in the development and refinement of the methodology of the experiments.

## Conflict of Interest

The authors declare that the research was conducted in the absence of any commercial or financial relationships that could be construed as a potential conflict of interest.
